# Collateral damage from violent incidents: human costs of polio immunization

**DOI:** 10.2471/BLT.25.293307

**Published:** 2025-06-10

**Authors:** Kamran Badizadegan, Kimberly M Thompson

**Affiliations:** a Kid Risk, Inc., 7512 Dr Phillips Boulevard #50-523, Orlando, FL 32819, United States of America.

## Abstract

**Objective:**

To provide observational statistics on reported violent incidents, deaths and injuries associated with polio immunization activities in the context of global polio eradication efforts.

**Methods:**

We made a systematic search of registries dedicated to documenting violence against health workers, as well as online news reports related to targeted attacks against polio immunization activities.

**Findings:**

We identified 362 violent incidents between 1998 and 2024 that reported 359 deaths, 358 non-fatal injuries and 179 kidnappings associated with polio immunization activities. Incidents included attacks on immunization facilities, workers, support staff (for example, security and transportation) and/or vaccine recipients, which also sometimes involved family members of vaccinees or uninvolved bystanders. The reported incidents occurred in 14 countries. The two countries still endemic for wild poliovirus transmission as of 2025 accounted for 85% (607/717) of the total deaths and injuries: Pakistan 69% (497/717) and Afghanistan 15% (110/717). Of the deaths and injuries reported, 47% (404/857) were vaccine delivery personnel, including individuals identified as volunteers, workers or staff and 28% (236/857) were security personnel. The total number of violent incidents peaked in 2014 (51 incidents, 64 deaths and 56 injuries), followed by 2024 (42 incidents, 40 deaths and 63 injuries), which are double the average of the preceding 5 years.

**Conclusion:**

We found substantial human costs associated with the polio immunization activities conducted to achieve the goal of global polio eradication. Efforts are needed to find effective solutions to protect individuals on the frontline of polio immunization activities, particularly in places where the risks are highest.

## Résumé

**Objectif:**

Fournir des statistiques observationnelles sur les incidents violents, décès et blessures signalés associés aux activités de vaccination contre la poliomyélite dans le contexte des efforts mondiaux d’éradication de cette maladie.

**Méthodes:**

Nous avons effectué une recherche systématique des registres dédiés à la documentation de la violence ciblant les travailleurs de la santé, ainsi que des dépêches en ligne relatives aux attaques ciblant les activités de vaccination contre la poliomyélite.

**Résultats:**

Nous avons identifié 362 incidents violents entre 1998 et 2024 qui ont fait état de 359 décès, de 358 blessures non mortelles et de 179 enlèvements associés aux activités de vaccination contre la poliomyélite. Ces incidents comprenaient des attaques contre des infrastructures de vaccination, des personnes qui y travaillent, du personnel de soutien associé (comme en matière de sécurité et de transport) et/ou des personnes vaccinées, qui impliquaient aussi parfois des membres de la famille des personnes vaccinées ou des passants non impliqués. Les incidents signalés se sont produits dans 14 pays. Les deux pays où la transmission du poliovirus sauvage était encore endémique en 2025 représentaient 85% (607/717) du total des décès et des blessures: 69% au Pakistan (497/717) et 15% en Afghanistan (110/717). Parmi les victimes recensées, 47% (404/857) étaient chargées de la distribution des vaccins, y compris des bénévoles, des travailleurs ou des employés, et 28% (236/857) étaient des membres du personnel de sécurité. Le nombre total d’incidents violents a culminé en 2014 (51 incidents, 64 morts et 56 blessés), ainsi qu’en 2024 (42 incidents, 40 morts et 63 blessés), ce qui représente le double de la moyenne des cinq années précédentes.

**Conclusion:**

Nous avons constaté que les activités de vaccination contre la poliomyélite menées pour atteindre l’objectif d’éradication de cette maladie dans le monde entraînaient des coûts humains considérables. Des efforts s'imposent pour trouver des solutions efficaces afin de protéger les personnes en première ligne des activités de vaccination contre la poliomyélite, en particulier dans les endroits où les risques sont les plus élevés.

## Resumen

**Objetivo:**

Proporcionar estadísticas observacionales sobre los incidentes violentos, muertes y lesiones asociados con las actividades de inmunización contra la poliomielitis en el contexto de los esfuerzos mundiales para su erradicación.

**Métodos:**

Se realizó una búsqueda sistemática en registros dedicados a documentar la violencia contra el personal sanitario, así como en reportes periodísticos en línea relacionados con ataques dirigidos a actividades de inmunización contra la poliomielitis.

**Resultados:**

Se identificaron 362 incidentes violentos entre 1998 y 2024 que reportaron 359 muertes, 358 lesiones no mortales y 179 secuestros asociados a actividades de inmunización contra la poliomielitis. Los incidentes incluyeron ataques a instalaciones de inmunización, personal vacunador, personal de apoyo (por ejemplo, seguridad y transporte) o personas receptoras de la vacuna, que en algunos casos también involucraron a familiares de las personas vacunadas o a transeúntes no implicados. Los incidentes reportados ocurrieron en 14 países. Los dos países que aún presentaban transmisión endémica de poliovirus salvaje en 2025 concentraron el 85% (607/717) del total de muertes y lesiones: Pakistán con el 69% (497/717) y Afganistán con el 15% (110/717). Del total de víctimas reportadas, el 47% (404/857) correspondió a personal encargado de la entrega de vacunas, incluidas personas identificadas como voluntarias, trabajadoras o integrantes del equipo, y el 28% (236/857) a personal de seguridad. El número total de incidentes violentos alcanzó su punto máximo en 2014 (51 incidentes, 64 muertes y 56 lesiones), seguido de 2024 (42 incidentes, 40 muertes y 63 lesiones), lo cual representa el doble del promedio registrado en los cinco años anteriores.

**Conclusión:**

Se observaron costos humanos sustanciales asociados a las actividades de inmunización contra la poliomielitis realizadas para alcanzar la meta de erradicación mundial. Se requieren esfuerzos urgentes para encontrar soluciones eficaces que protejan a las personas que están en la primera línea de estas actividades, en particular en los contextos donde los riesgos son más elevados.

## ملخص

الغرض تقديم إحصاءات رصدية حول حوادث العنف والوفيات والإصابات التي تم الإبلاغ عنها، والمرتبطة بأنشطة التطعيم ضد شلل الأطفال، في سياق الجهود العالمية للقضاء على شلل الأطفال.

الطريقة لقد أجرينا بحثًا منهجيًا في السجلات المُخصصة لتوثيق العنف ضد العاملين فيي المجال الصحي، بالإضافة إلى التقارير الإخبارية الإلكترونية المتعلقة بالهجمات المُستهدفة ضد أنشطة التطعيم لشلل الأطفال.

النتائج توصلنا لاكتشاف 362 حادثة عنف بين عامي 1998 و2024، أسفرت عن 359 حالة وفاة، و358 إصابة غير مميتة، و179 حالة اختطاف مرتبطة بأنشطة التطعيم ضد شلل الأطفال. وشملت الحوادث هجمات على مرافق التطعيم، والعاملين، وموظفي الدعم (مثل الأمن والنقل)، و/أو متلقي اللقاح، والتي شملت أحيانًا أفرادًا من عائلات المتلقين للتطعيم أو مشاهدين غير مشاركين. وقعت الحوادث التي تم الإبلاغ عنها في 14 دولة. لا تزال الدولتان الموبوءتان بانتشار فيروس شلل الأطفال البري حتى عام 2025 مسؤولتان عن %85 (607/717) من إجمالي الوفيات والإصابات: باكستان %69 (497/717) وأفغانستان %15 (110/717). من بين الضحايا الذين تم الإبلاغ عنهم، كان %47 (404/857) من موظفي توصيل اللقاح، بمن فيهم الأفراد الذين تم تحديدهم كمتطوعين أو عمال أو موظفين، و %28 (236/857) من أفراد الأمن. بلغ العدد الإجمالي للحوادث العنيفة ذروته في عام 2014 (51 حادثة، و64 حالة وفاة، و 56 إصابة)، يليه عام 2024 (42 حادثة، و40 حالة وفاة، و63 إصابة)، وهو ضعف متوسط السنوات الخمس السابقة.

الاستنتاج لقد وجدنا تكاليف بشرية كبيرة مرتبطة بأنشطة التطعيم ضد شلل الأطفال، والتي أجريت لتحقيق هدف القضاء على شلل الأطفال العالمي. هناك حاجة للجهود لإيجاد حلول فعالة لحماية الأفراد في الخطوط الأمامية لأنشطة التطعيم ضد شلل الأطفال، وخاصة في الأماكن التي تكون فيها المخاطر أعلى ما يمكن.

## 摘要

目的 基于在为消灭脊灰问题而开展的全球脊灰免疫接种活动中报告的暴力事件、死亡和受伤情况，提供观察统计数据。

方法 我们系统地搜索了专用于记录卫生工作者所遭遇暴力事件的登记册，以及与针对脊灰免疫接种活动所发起攻击相关的在线新闻报道。

结果 我们发现，在 1998 至 2024 年期间报告的 362 起与脊灰免疫接种活动相关的暴力事件中，共有 359 人死亡，358 人受到了非致命伤害，并且有 179 人遭到了绑架。在暴力事件中遭受攻击的对象包括免疫接种机构、卫生工作者、支援人员（例如负责安保和运输事务的人员）和/或疫苗接种者（有时还包括接种者的家人或无辜受殃者）。据报告，共有 14 个国家发生了此类暴力事件。以下两个国家还出现了野生脊灰病毒地方性传播的情况，截至 2025 年，其死亡和受伤总人数占 85% (607/717)：巴基斯坦占 69% (497/717)，阿富汗占 15% (110/717)。在报告的伤亡人数中，疫苗交付相关人员（包括被视为志愿者、卫生工作者或支援人员的人员）占 47% (404/857)，安保人员占 28% (236/857)。2014 年发生的暴力事件总数最多（51 起暴力事件，共有 64 人死亡和 56 人受伤）；其次是 2024 年（42 起暴力事件，共有 40 人死亡，63 人受伤），达到了前 5 年平均数的两倍。

结论 我们发现，为实现在全球范围内消灭脊灰问题这一目标而开展的脊灰免疫接种活动会造成大量人员伤亡。仍需不断努力制定有效的解决方案以保护参与脊灰免疫接种活动的一线人员，特别是在风险最高的地区。

## Резюме

**Цель:**

Представить статистические данные наблюдений о зарегистрированных случаях насилия, смерти и травматизма, связанных с мероприятиями по вакцинации от полиомиелита, в контексте глобальных усилий по ликвидации полиомиелита.

**Методы:**

Авторы провели систематический поиск по реестрам, специализирующимся на документировании случаев насилия в отношении работников здравоохранения. Дополнительно были изучены новостные публикации в сети Интернет, освещающие целенаправленные акты насилия против мероприятий по вакцинации от полиомиелита.

**Результаты:**

Авторы выявили 362 случая насилия в период между 1998 и 2024 годами, в которых сообщалось о 359 смертях, 358 случаях несмертельных травм и 179 похищениях, связанных с мероприятиями по вакцинации от полиомиелита. Происшествия включали нападения на пункты вакцинирования, работников, помощников (например, охрану и водителей) и/или на вакцинируемых, в том числе время от времени на членов семьи вакцинируемых или даже на посторонних лиц, случайно присутствовавших при вакцинации. Сообщения о происшествиях были зарегистрированы в 14 странах. Две страны все еще остаются эндемичными по передаче дикого вируса полиомиелита по состоянию на 2025 год, и в них наблюдаются 85% (607/717) от общего количества смертей и травм: Пакистан 69% (497/717) и Афганистан 15% (110/717). Среди зарегистрированных жертв 47% (404/857) составили лица, доставлявшие вакцину, в том числе те, кто были идентифицированы как волонтеры, работники или персонал, и 28% (236/857) – сотрудники охранной службы. Наибольшее количество случаев насилия имело место в 2014 году (51 инцидент, 64 случая смерти и 56 травм), а затем в 2024 году (42 инцидента, 40 случаев смерти и 63 травмы), что вдвое больше, чем средние показатели в предыдущие 5 лет.

**Вывод:**

Авторы обнаружили, что мероприятия по вакцинации от полиомиелита, проводимые в рамках всемирных усилий по ликвидации данного заболевания, сопряжены со значительными гуманитарными последствиями. Необходимы усилия по поиску эффективных решений для защиты лиц, находящихся на передовой линии работ по вакцинации от полиомиелита, особенно в регионах с наибольшими рисками.

## Introduction

In the mid-1980s, on the heels of the global eradication of smallpox^1^ and with substantial early gains of the Expanded Programme on Immunization,^2^ some public health leaders increasingly viewed polio eradication as all but inevitable^3^. In a consensus-building effort organized by the Task Force for Child Survival, which included 60 global health leaders, polio eradication topped the list of lifesaving activities to promote children’s health.^4^ Shortly thereafter, in May 1988, the World Health Assembly (WHA) adopted a resolution to eradicate polio by the year 2000.^5^ Notably, after adopting “the eradication of poliomyelitis and the alleviation of its consequences” as a primary goal in 1979,^6^ Rotary International provided substantial global political and financial support for these decisions, along with a so-called army of dedicated volunteers committed to the cause.

Poliovirus vaccines prevented significant numbers of paralysis cases^7^ and deaths^2^ following their widespread adoption and use in national and international immunization activities. However, 37 years after the WHA resolution to eradicate polio, what was once considered inevitable remains a more challenging and complicated task than originally imagined, as demonstrated by many changes in eradication goals, strategies and tactics.^8^ The Global Polio Eradication Initiative continues to struggle with ending wild poliovirus transmission in the two remaining endemic countries (Pakistan and Afghanistan) and with the transmission of circulating vaccine-derived polioviruses, alternatively referred to as variant polioviruses.^9^ Type 2 circulating vaccine-derived polioviruses reported after ending the use of type 2 oral poliovirus vaccines (OPV) for preventive immunizations in 2016 provided evidence of the challenges of stopping OPV.^10^^,^^11^

Supplementary immunization activities that aim to achieve and maintain polio eradication face real human costs as vaccine providers, support staff and others may come under targeted attack during immunization activities. With continued delays in achieving polio eradication, evolving strategies of the Global Polio Eradication Initiative, the changing global geopolitical landscape, and growing mistrust of vaccines and vaccine hesitancy, attacks on teams undertaking polio supplementary immunization activities have intensified in the endemic countries.^8^^,^^12^^–^^14^ Although violent incidents occur relatively infrequently given the large number of polio supplementary immunization activities conducted globally,^8^ the incidents extend over a period of two decades and the risks vary substantially by country. An article on violent incidents in Pakistan reported that more than 200 people were killed while working on polio immunization between 2012 and 2021.^15^ Earlier incident reports or commentaries discussed violent incidents associated with polio immunization,^16^^–^^22^ but did not synthesize this information into a comprehensive database or analysis.

In this study, we aimed to collect and synthesize the available data on violence related to polio immunization from all sources to characterize the global numbers of reported violent incidents, deaths and injuries associated with these activities between 1998 and 2024. Although the global commitment to polio eradication was launched in 1988,^5^ intensification of polio immunization activities began in the late 1990s^8^ and we could not identify any specific incidents for inclusion before 1998.

## Methods

We define violent incidents associated with polio immunization activities as attacks on polio vaccine facilities or property (for example, vehicles), workers, volunteers, support staff (for example, security and transportation personnel and observers), and/or vaccine recipients and their families for which we can identify the location and date when the event occurred. We separately counted incidents that occurred on the same date in different locations, even though these incidents may represent coordinated events.

To develop a comprehensive database of reported violent incidents associated with polio immunization, we searched publicly available registries of violent incidents for reports that potentially involved polio immunization efforts.^23^^–^^28^ We also searched internet pages reporting such incidents (Table 1). For downloadable registries,^23^^,^^24^^,^^28^ we extracted the data into a spreadsheet and identified polio-related entries using a word search for “polio”. For other registries,^25^ we did keyword searches for “polio” before downloading the retrieved entries into a spreadsheet. Other candidate data sources^26^^,^^27^ did not include usable data for this study.

**Table 1:** Data sources and selection

Source	Source reporting period	No.			Reason(s) for exclusion of source entries
		Relevant entries	Incidents included	Unique incidents^a^	
Global Terrorism Database^23^	1970–2020	192	192	140	None
CDC Foundation^29^	2001–2024	315	89	70	Unrelated events (e.g. car crashes and medical emergencies)
South Asia Terrorism Portal ^25^	2020–2024	43	42	6	Duplicate record
Aid Worker Security Database^24^	1997–2024	41	41	7	None
Google search	Before 1970–2024	> 1200	105	31	Duplicate reports, unrelated search results
@KhorasanDiary tweets	2022–2024	32	32	1	None
Surveillance System for Attacks on Health Care^28^	2017–2024	1	1	0	None
**Total**	**NA**	**> 1822**	**501**	**255**	**NA**

CDC: US Centers for Disease Control and Prevention; NA: not applicable.

^a^ We identified the same incident in more than one source on many occasions. To avoid double counting, we counted each incident only once. The number of unique incidents is the number of incidents that we identified only from the source mentioned.

We retrieved internet pages from Google using the search string: (“polio gunman OR gunmen OR killed OR injured OR kidnapped”). Based on Google search rules, this search resulted in pages that include the word “polio” in addition to one or more of the subsequent search terms. We did a broad Google search, then a specific search within the websites: tolonews.com (TOLOnews, Kabul, Afghanistan) and dawn.com (Dawn Media Group, Karachi, Pakistan), which regularly report the news from Afghanistan and Pakistan, respectively. To ensure that we captured older reports in our Google searches, we re-did every search with time modifiers to limit results to pages last updated before 2010, 2005, 2000 and 1995. In addition, we retrieved tweets including the word “polio” by @khorasandiary on X.com® (X Corp., Bastrop, USA) as the Khorasan Diary (Islamabad, Pakistan) often tweets polio-related police reports that include violent incidents in Afghanistan and Pakistan. We manually screened polio-related tweets as well as the top 50 Google search results per search for relevance and data extraction. For incidents with more than one entry in Google, we recorded only one representative source for reference in the data set. We did not track the number of excluded Google search duplicates given the large number of entries, most of which appeared automatically generated from another news source.

Lastly, we contacted the US Centers for Disease Control and Prevention (CDC) Foundation, which manages the Bob Keegan Heroes Fund, to request information about incidents associated with requests for financial support to compensate victims and/or their families from polio volunteer- or work-related injuries.^29^

Combining information from all of the above data sources, we developed a spreadsheet that included the incident date, country, specific location (province, district and/or city, when available) and a brief description of each incident. Consistent with South Sudan gaining independence in mid-2011, we recorded all reported incidents that occurred in areas now part of South Sudan as Sudan before mid-2011. We used a composite key consisting of incident date, location and incident description to ensure unique identification of incidents. For each unique incident, we recorded the reported number of people killed (by sex, if reported) or injured, and if the incident report noted kidnapping, deaths and injuries among children and/or property damage. We recorded the role of each casualty as: (i) volunteer; (ii) worker; (iii) World Health Organization (WHO) or United Nations (UN) staff; (iv) security personnel or police officer; (v) vaccine recipient and/or family member; (vi) bystander; or (vii) unknown (that is, unreported) using the terminology in the source data. We recorded assailants killed during the attack when reported, but did not otherwise focus on assailant deaths and injuries. We summarized the data by total numbers of incidents by year and country. We coded each individual casualty once, as a death for a reported fatality, even if the report also stated the individual experienced injury. Thus, our summary statistics bias towards severity.

## Results

**Fig. 1:**
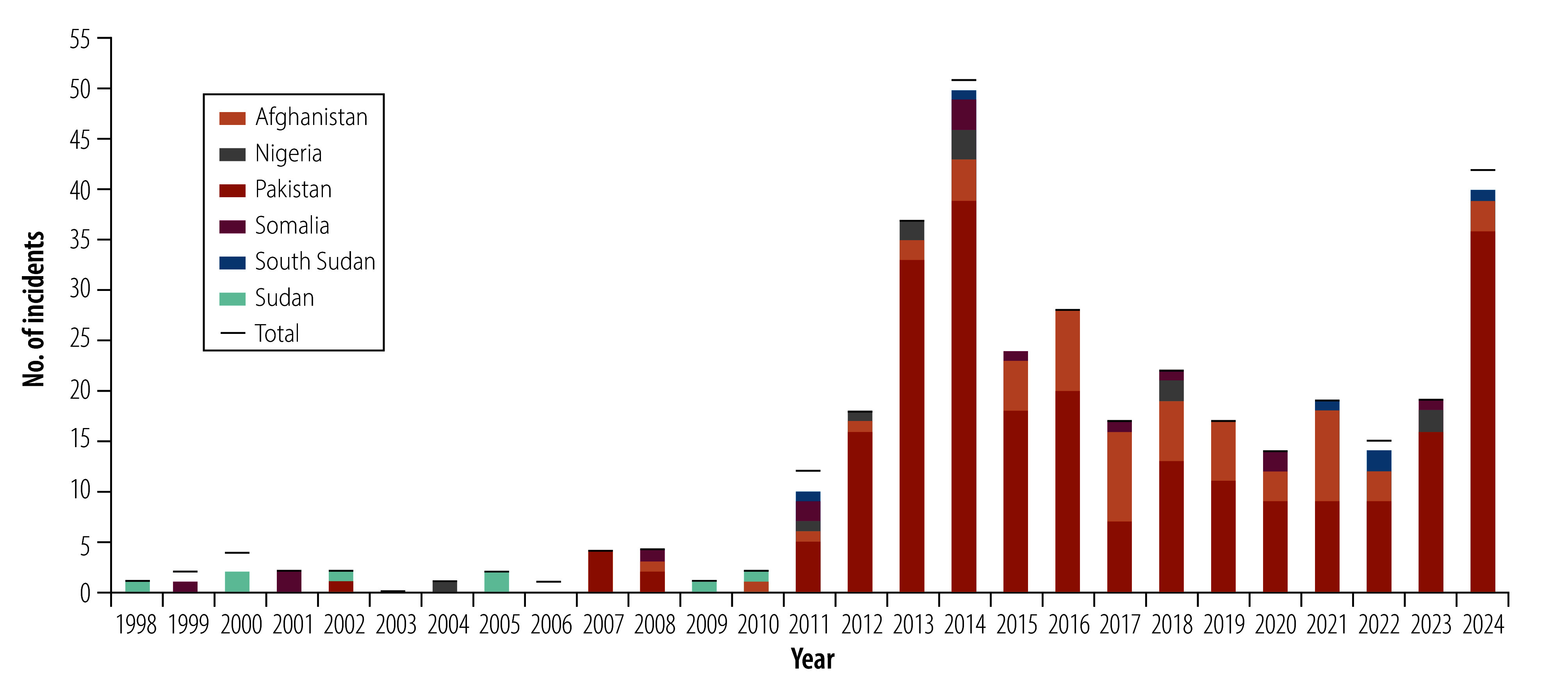
Incidents of violence associated with polio immunization activities, by year and country

Note: Black horizontal dashes show the total number of incidents, i.e. 1999 Angola (one incident), 2000 Democratic Republic of the Congo (one incident) and Angola (one incident), 2006 India (one incident), 2011 Central African Republic (one incident) and India (one incident), 2014 Central African Republic (one incident) and Syrian Arab Republic (one incident), 2019 Mali (one incident), 2022 Burkina Faso (one incident), 2024 West Bank and Gaza Strip (one incident). Coloured bars show the numbers of incidents for the six countries that reported more than two incidents between 1998 and 2024.

We identified 362 reported violent incidents associated with polio immunization between 1998 (month and day of first incident not reported) and 31 December 2024. Fig. 1 shows the total number of incidents per year and by country that reported more than two total incidents over the time period (Afghanistan, Nigeria, Pakistan, Somalia, South Sudan and Sudan). Incidents ranged from 0 incidents reported in 2003 to 51 incidents in 2014. The data included incident reports from eight other countries not shown in Fig. 1, which reported one incident (Democratic Republic of the Congo in 2000, Syrian Arab Republic in 2014, Mali in 2019, Burkina Faso in 2022, and West Bank and Gaza Strip in 2024); or two total incidents (Angola in 1999 and 2000, India in 2006 and 2011 and Central African Republic in 2011 and 2014). Of note, 2024 had the second highest number of total incidents (42 incidents); more than double the number of incidents than the average of the preceding 5 years.

**Fig. 2:**
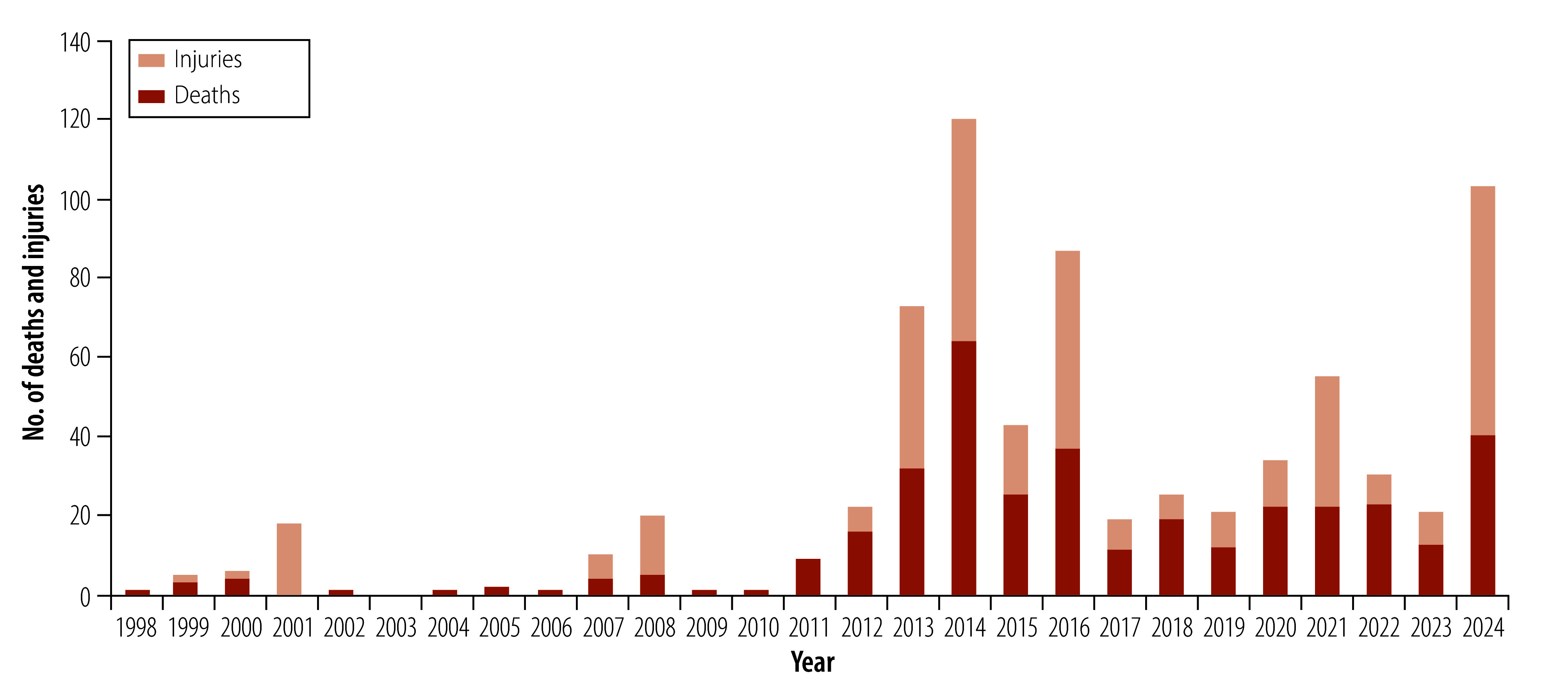
Deaths and injuries associated with polio immunization activities, 1998–2024

**Fig. 3:**
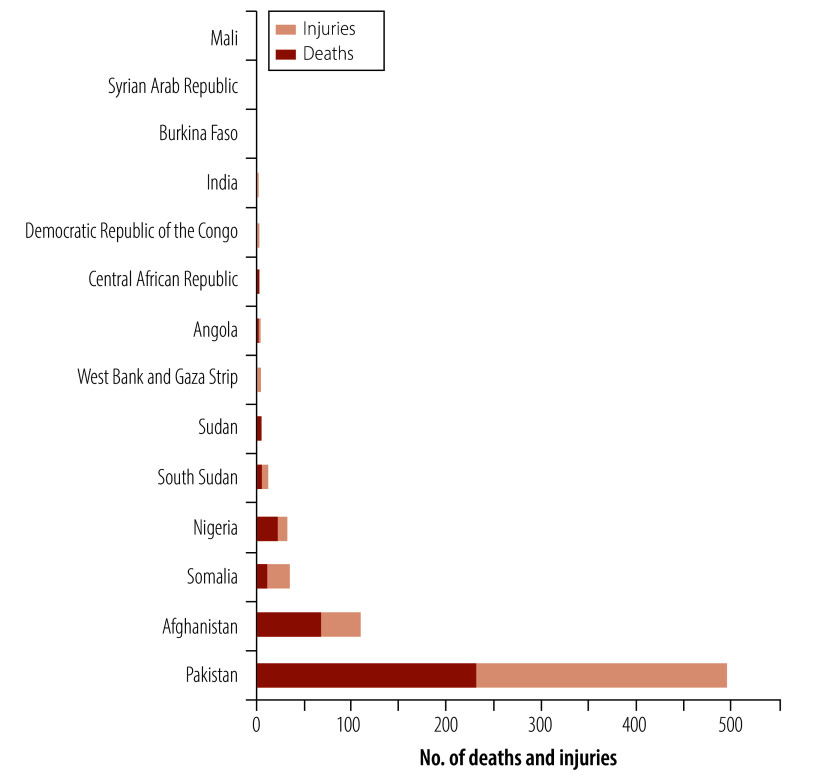
Deaths and injuries associated with polio immunization activities, by country, 1998–2024

Fig. 2 shows the total number of reported deaths and injuries by year and Fig. 3 shows the total numbers by country. Pakistan and Afghanistan reported the highest number of deaths and injuries, accounting for 85% (607/717) of the total deaths and injuries: Pakistan 69% (497/717) and Afghanistan 15% (110/717). Overall, 359 deaths have been reported in 218 violent incidents, and 358 non-fatal injuries in 121 violent incidents associated with polio immunization efforts since 1998 (Table 2). Among the deaths and injuries were 18 individuals in 10 incidents who were reported as being a child or younger than 18 years of age at the time of the attack. Most of the fatalities (55%; 197/359) were males, although for a substantial proportion of fatalities (31%; 112/359), sex was not reported. Overall, reports included similar numbers of fatalities (359) and non-fatal injuries (358), which may reflect some bias towards reporting fatalities. We identified 50 incidents that reported 179 individuals kidnapped. Most of these incidents were resolved with no reported injury or death. However, incidents involving kidnapping included 18 non-exclusive events resulting in 22 deaths and 17 injuries that we included in the statistics for deaths and injuries.

**Table 2:** Injuries, deaths and incidents, by type

Variable	Deaths and injuries	Incidents
**Total**	857	362
**Fatalities**	359	218
Sex of individuals who died		
Male	197	140
Female	50	32
Not specified	112	57
**Non-fatal injuries**	358	121
Incident included reported fatalities	208	50
Incident with no reported fatalities	150	71
**Kidnappings**	179	49
With injury and/or death^a^	39	20
Kidnapping only (no injury and/or death)	140	29
**Deaths and injuries < 18 years or reported as a child^a^**	18	10

^a^ Already counted in fatalities and/or injuries.

As coded based on the information provided in incident reports and registries, the roles of the victims of violent incidents are summarized in Table 3. Of all the injuries or deaths, 40% (340/857) were polio immunization workers and volunteers (that is, individuals with primary responsibility for vaccinating children in supplementary immunization activities). Police and other security personnel accounted for 28% (236/857) of the deaths and injuries. The specific roles of 14% (118/857) of the injured or dead were not reported.

**Table 3:** Reported roles of the people targeted

Role	No. (%) (*n* = 857)
Polio worker or volunteer, not otherwise specified	340 (40)
WHO or UN staff assigned to polio campaign	64 (7)
Police or other security personnel	236 (28)
Family member of vaccinee or civilian bystander	99 (12)
Not specified	118 (14)

UN: United Nations; WHO: World Health Organization.

Of the 362 reported violent incidents associated with polio immunization activities, 318 (88%) reported fatalities, injuries and/or kidnappings; while no deaths and injuries associated with polio immunization activities were reported for the remaining 44 (12%) incidents (Table 4). Of these 44 incidents, no deaths and injuries, or property damage were reported in 22 and property damage only was reported in 16 incidents. We also noted incidents that reported 11 assailant fatalities: three among the 362 incidents with non-assailant deaths and injuries resulted in five assailant deaths, and three incidents of thwarted attacks resulted in six assailant deaths. We did not code data on assailant injuries or arrests but noted occasional reports of such injuries in some of the incident descriptions.

**Table 4:** Outcomes of the reported incidents

Outcome	No. (%) of incidents (*n* = 362)
Deaths and injuries reported	318 (88)
No deaths and injuries, or property damage (resolved)	22 (6)
Property damage only (no deaths and injuries)	16 (4)
Insufficient information to code and classify deaths and injuries	6 (2)
Assailant fatalities reported	6 (2)
- Incidents with deaths and injuries	3 (1)
- With no deaths and injuries (resolved)	3 (1)

## Discussion

Every life lost in the line of duty is a tragic loss, and acknowledging the people killed, injured and kidnapped while supporting polio immunization efforts highlights the human costs associated with polio immunization. A systematic documentation of the extent and human cost of these incidents globally has been lacking. Our results clearly show the occupational and volunteer risks associated with polio immunization efforts in a small number of countries, particularly in Afghanistan and Pakistan, and particularly associated with supplementary immunization activities. These risks contrast with the experiences in other countries in which polio supplementary immunization activities and other immunization efforts occur peacefully.

Several limitations may make our analysis incomplete. First, we cannot assess the extent to which our integrated database provides a comprehensive list of incidents. The Aid Worker Security Database,^24^ which provides data specific to staff members of various UN organizations, does not include volunteers and police officers, among others. The Bob Keegan Polio Eradication Heroes Fund^29^ provides financial assistance to polio volunteer workers and their families but does not cover non-volunteers (for example, UN staff or security personnel). Other databases^23^^,^^25^^–^^28^ may not capture polio-related incidents, may contain reporting inaccuracies or may not label some of the incidents as polio-related. These factors limited our ability to identify all relevant incidents. A 2023 article discussed violence against polio workers in Pakistan only,^15^ but did not provide a detailed description of the method or sources. WHO reported a significant number of violent incidents against health workers in Sudan between April 2023 and July 2024 resulting in 55 deaths and 104 injuries.^30^ Although these statistics likely included polio workers, given the presence of circulating vaccine-derived polioviruses during the same period, we could not find specific details about these incidents from WHO or others to make any attributions. Other publications reported on a limited number of specific incidents or provided editorial perspectives.^16^^–^^21^ By combining multiple sources of data and independently searching reports, we sought to develop the most comprehensive database available to date. However, we recognize that the database probably misses or incompletely characterizes some incidents because of the incomplete nature of primary sources.

Second, we could not identify the number of individuals engaged in different activities that would provide denominators that might allow us to characterize the risk to polio workers in different countries. Based on the available data, our analysis suggests substantially higher risks associated with polio immunization activities in health workers in Afghanistan and Pakistan than in other countries.

Third, we could not identify all potentially relevant events. For example, although we found evidence of some thwarted attacks in the reported data as noted earlier, we recognize that prevented events generally do not get counted. In addition, we did not include some reported incidents for which we could not find sufficiently detailed reports. For instance, we could not find sufficient information for at least 11 non-fatal incidents in Pakistan of threats, harassment, beating, rape and/or injury reported elsewhere.^15^ Recognizing that the Global Polio Eradication Initiative places emphasis on the key role of women delivering immunization on the frontline,^31^ our observation of a substantial number of female fatalities (Table 2) provides an indication of the risks that they face in some countries. However, a considerable number of incident reports gave no information about the victims’ sex or other demographic characteristics. Finally, we suspect, but cannot verify, under-reporting of violent incidents involving women, specifically incidents involving sexual assault, which local and national sources may track, such as those incidents described elsewhere.^15^ A high profile news report detailed gang rape of a female polio immunization worker in Pakistan who did not initially report the assault as rape, most likely from fear of being expelled from her home or threats to her life after being accused of dishonouring the family.^32^ This recent case provides one of the rare instances that mentions sexual assault in violent incidents against polio teams.

Previous studies suggest multiple complex factors drive the large number of incidents in Afghanistan and Pakistan, the two remaining countries that are endemic for wild poliovirus. Our results show substantial increases in incidents, deaths and injuries of polio immunization workers in Pakistan after the US Central Intelligence Agency used an immunization campaign as part of its efforts to identify the location of Osama Bin Laden in July 2011.^33^ Researchers analysed this event and the issues of conflict and militancy, negative propaganda against vaccines, and vaccine bans as challenges to polio immunization in Pakistan.^34^ Other studies and anecdotal reports suggest that public perception of polio and poliovirus vaccines is based on misinformation and negative sentiments.^34^^–^^36^ In a study of 241 parents or guardians of children younger than 5 years of age in Multan (Punjab, Pakistan) who repeatedly refused poliovirus vaccines, just over half the participants viewed poliovirus vaccines as harmful and about a sixth considered them unnecessary.^36^ Similar sentiments were reported in 2020 from Karachi (Sindh province), Pishin (Baluchistan province) and Bajaur district (Khyber Pakhtunkhwa) in Pakistan.^35^ The study reported a significant increase in the proportion of participants who responded that they ever refused to give polio drops to their child in Karachi and Pishin in 2020 compared with results obtained in 2012.^35^ Another hypothesis for the increase in OPV refusal may reflect preferences for the injectable inactivated poliovirus vaccine, particularly given its use in high-income countries and it being described as safer than OPV.^37^ The injectable inactivated poliovirus vaccine was introduced in Pakistan into some supplementary immunization activities in 2014 and routine immunization in 2015. Regardless of the underlying causes, the general increase in the use of violence for political and/or economic gains in endemic countries and regions with a high incidence of circulating vaccine-derived polioviruses continues to pose substantial challenges for polio eradication efforts.^38^^,^^39^

No health worker should face intentional acts of violence in the course of providing care. The human costs presented here reflect tragic losses for individuals and their families, as well as collateral damage (that is, measurable economic costs) for polio immunization efforts. In addition, violence against polio immunization teams conducting supplementary immunization activities frequently leads to early termination or complete cancellation of the activities. These activities are an essential requirement for successfully stopping and preventing the transmission of polioviruses in some countries. While security personnel assigned to immunization teams prevent some acts of violence, the large number of deaths and injuries these staff experience demonstrates the limited impact of deterrence and the extent to which some assailants appear willing to die for their causes. Delays in achieving the end of poliovirus transmission in Afghanistan and Pakistan suggest the need to find peaceful strategies for communities to safely and effectively deliver vaccines.

The frontline workers who accept real risks to deliver poliovirus vaccines remain true heroes of polio eradication efforts. While we recognize that reporting cumulative numbers runs the risk of normalizing the risks or failing to recognize the individual toll they represent, we hope that our analysis will lead to greater acknowledgement of the dangers faced by people on the frontline of immunization and to finding more effective solutions to protect them.
